# Pyruvate sensitizes pancreatic tumors to hypoxia-activated prodrug TH-302

**DOI:** 10.1186/s40170-014-0026-z

**Published:** 2015-01-29

**Authors:** Jonathan W Wojtkowiak, Heather C Cornnell, Shingo Matsumoto, Keita Saito, Yoichi Takakusagi, Prasanta Dutta, Munju Kim, Xiaomeng Zhang, Rafael Leos, Kate M Bailey, Gary Martinez, Mark C Lloyd, Craig Weber, James B Mitchell, Ronald M Lynch, Amanda F Baker, Robert A Gatenby, Katarzyna A Rejniak, Charles Hart, Murali C Krishna, Robert J Gillies

**Affiliations:** Department of Imaging and Metabolism, H. Lee Moffitt Cancer Center and Research Institute, Tampa, FL 33612 USA; Department of Radiology, H. Lee Moffitt Cancer Center and Research Institute, Tampa, FL 33612 USA; Department of Integrated Mathematical Oncology, H. Lee Moffitt Cancer Center and Research Institute, Tampa, FL 33612 USA; Analytic Microscopy Core Facility, H. Lee Moffitt Cancer Center and Research Institute, Tampa, FL 33612 USA; Arizona Cancer Center, College of Medicine, University of Arizona, Tucson, AZ 85724 USA; Department of Physiology, College of Medicine, University of Arizona, Tucson, AZ 85724 USA; Hematology/Oncology Section, College of Medicine, University of Arizona, Tucson, AZ 85724 USA; Center for Cancer Research, National Cancer Institute, Bethesda, MD 20892 USA; Threshold Pharmaceutical, San Francisco, CA 94080 USA; Department of Oncologic Sciences, University of South Florida, Tampa, FL 33612 USA

**Keywords:** Hypoxia, Hypoxia-activated prodrugs, TH-302, Tumor microenvironment, Metabolism, Pancreatic cancer, Functional imaging, Computational modeling

## Abstract

**Background:**

Hypoxic niches in solid tumors harbor therapy-resistant cells. Hypoxia-activated prodrugs (HAPs) have been designed to overcome this resistance and, to date, have begun to show clinical efficacy. However, clinical HAPs activity could be improved. In this study, we sought to identify non-pharmacological methods to acutely exacerbate tumor hypoxia to increase TH-302 activity in pancreatic ductal adenocarcinoma (PDAC) tumor models.

**Results:**

Three human PDAC cell lines with varying sensitivity to TH-302 (Hs766t > MiaPaCa-2 > SU.86.86) were used to establish PDAC xenograft models. PDAC cells were metabolically profiled *in vitro* and *in vivo* using the Seahorse XF system and hyperpolarized ^13^C pyruvate MRI, respectively, in addition to quantitative immunohistochemistry. The effect of exogenous pyruvate on tumor oxygenation was determined using electroparamagnetic resonance (EPR) oxygen imaging. Hs766t and MiaPaCa-2 cells exhibited a glycolytic phenotype in comparison to TH-302 resistant line SU.86.86. Supporting this observation is a higher lactate/pyruvate ratio in Hs766t and MiaPaCa xenografts as observed during hyperpolarized pyruvate MRI studies *in vivo*. Coincidentally, response to exogenous pyruvate both *in vitro* (Seahorse oxygen consumption) and *in vivo* (EPR oxygen imaging) was greatest in Hs766t and MiaPaCa models, possibly due to a higher mitochondrial reserve capacity. Changes in oxygen consumption and *in vivo* hypoxic status to pyruvate were limited in the SU.86.86 model. Combination therapy of pyruvate plus TH-302 *in vivo* significantly decreased tumor growth and increased survival in the MiaPaCa model and improved survival in Hs766t tumors.

**Conclusions:**

Using metabolic profiling, functional imaging, and computational modeling, we show improved TH-302 activity by transiently increasing tumor hypoxia metabolically with exogenous pyruvate. Additionally, this work identified a set of biomarkers that may be used clinically to predict which tumors will be most responsive to pyruvate + TH-302 combination therapy. The results of this study support the concept that acute increases in tumor hypoxia can be beneficial for improving the clinical efficacy of HAPs and can positively impact the future treatment of PDAC and other cancers.

**Electronic supplementary material:**

The online version of this article (doi:10.1186/s40170-014-0026-z) contains supplementary material, which is available to authorized users.

## Background

Tumor hypoxia, a routinely observed phenotype, contributes to chemo- and radioresistance, causing major therapeutic concern [1]. Due to extensive hypoxia, considerable effort has been devoted to harness its therapeutic potential. To exploit hypoxia, a class of hypoxia-activated prodrugs (HAPs) has been developed to selectively target tumor cells residing in hypoxic niches [2,3]. The most common class of HAPs is based on 2-nitroimidazoles, exemplified by TH-302, which is activated by cytochrome P450 reductase and contains alkylating nitrogen mustard, Br-isophosphoramide, as a cytotoxic effector [4]. There are 12 clinical trials with TH-302 underway in a variety of cancers (e.g., pancreatic, sarcoma, breast, melanoma) (Additional file 1: Table S1).

The major predictor of TH-302 activity is the extent of tumor hypoxia [5]. We therefore hypothesized that *increasing tumor hypoxia* could improve TH-302 activity. Experimental evidence show that the most effective approach to decrease tumor oxygenation (pO_2_) is increasing the oxygen consumption rate (OCR) [6]. This was effective in RKO cells in which pharmacological inhibition of hypoxia-inducible factor 1-alpha (HIF-1α) with echinomycin increased OCR, decreased tumor pO_2_, and increased the activity of the HAP, tirapazamine [7]. While HIF-1α inhibition was effective at increasing OCR, pharmacological inhibition may induce chronic hypoxia that could promote further adaptation and possibly increased HAP side effects. A separate study demonstrated that [1-^13^C]pyruvate infusion during hyperpolarized MRI studies substantially decreased tumor pO_2_ and increased hypoxia in squamous cell carcinoma (SCC) tumors [8]. The effect was transient, with peak hypoxia observed at 30 min, returning to baseline within 5 h. Pyruvate also stimulates OCR across a panel of breast cancer cell lines [9], consistent with historical data that shows increased OCR in response to pyruvate (or lactate) in neurons and cardiac myocytes [10-12]. Because pyruvate 1) transiently decreases tumor pO_2_, 2) decreases tumor pO_2_ metabolically rather than pharmacologically, and 3) produces no detectable side effects, we hypothesize that pyruvate would be a successful adjuvant to enhance TH-302 efficacy.

A consequence of a desmoplastic stroma is poor perfusion, particularly in pancreatic ductal adenocarcinoma (PDAC). PDAC tumors are profoundly hypoxic in comparison to normal pancreatic tissue [13] which is a negative prognostic factor [14]. TH-302 is highly effective in preclinical pancreatic models [15] and has been effective in phase I/II trials in combination with gemcitabine (GEM), increasing progression-free survival of late-stage cancers by 2 months [16]. These encouraging results justified a phase III trial, which is ongoing (NCT01746979).

In the present study, we utilized PDAC models to demonstrate the ability of exogenous pyruvate to stimulate oxygen consumption *in vitro* and transiently decrease tumor pO_2_*in vivo*. The decrease in tumor pO_2_ correlated with improved TH-302 efficacy, demonstrated by significant tumor volume decreases and increased survival. We also identified potential imaging and histological biomarkers that can be used to predict responsive tumor types. These data support the concept that temporarily increasing tumor hypoxia can improve the efficacy of HAPs, including TH-302, against PDAC and other cancer types in the clinic.

## Methods

### Cell culture

SU.86.86 and Hs766t cells were obtained from Threshold Pharmaceuticals (Redwood City, CA, USA), and MiaPaCa-2 cells were obtained from American Type Cell Collection (ATCC, Manassas, VA, USA). The cells were maintained in RPMI-1640 (Life Technologies) supplemented with 10% fetal bovine serum (FBS) (HyClone) (SU.86.86) and Dulbecco’s modified Eagle’s medium-F12 (DMEM-F12) (Life Technologies) supplemented with 10% FBS (Hs766t and MiaPaCa-2). All three cell lines were resuscitated from low passage with all experiments carried out with cells of passage numbers less than 10 (SU.86.86) and 20 (Hs766t and MiaPaCa-2).

### Animal housing

All animal experiments were carried out at the Moffitt Cancer Center and National Cancer Institute in compliance with the Guide for the Care and Use of Laboratory Animal Resources [17]. Experimental protocols conducted at Moffitt Cancer Center were approved by the Institutional Animal Care and Use Committee, University of South Florida. Experimental protocols performed at the National Cancer Institute were approved by the Institutional Animal Care and Use Committee, National Cancer Institute.

### Tumor development and treatment

**Figure 1:**
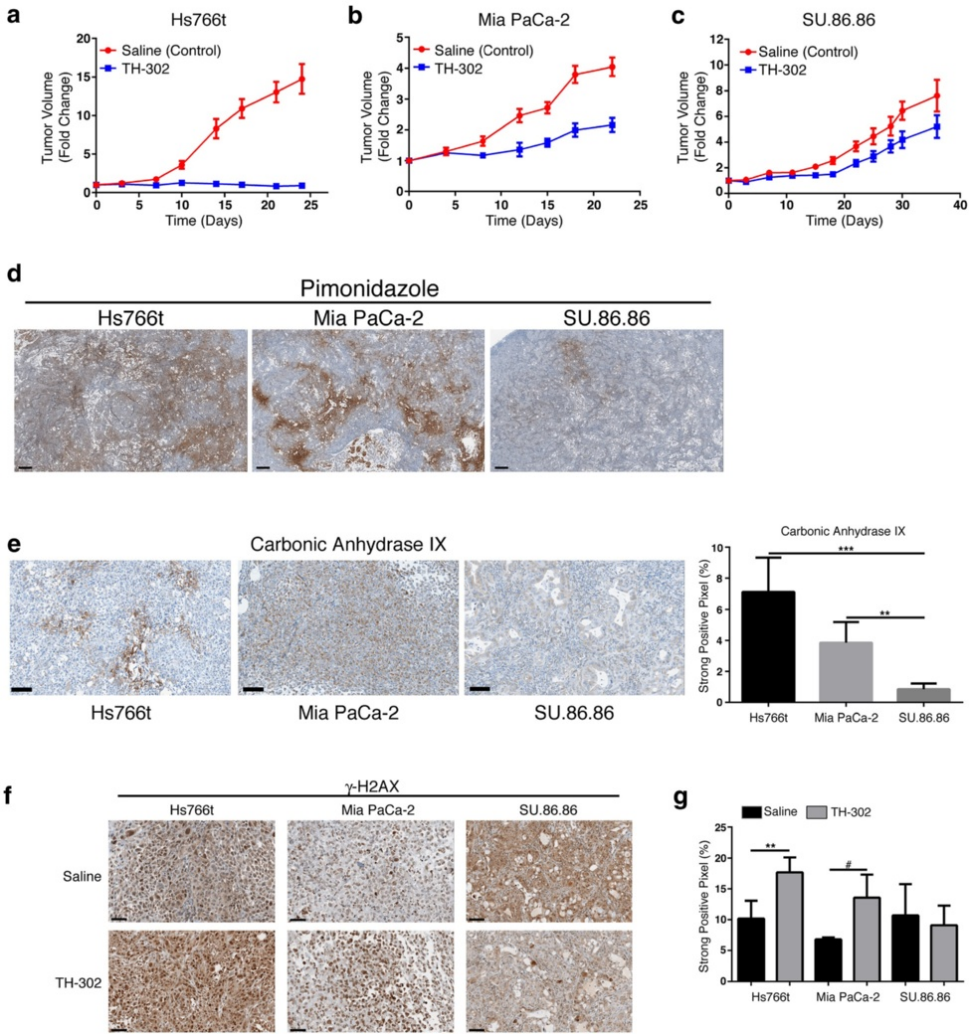
**Tumor hypoxia is main determinant for TH-302 Therapy.** **(a**-**c)** Monotherapy TH-302 efficacy in Hs766t **(a)**, MiaPaCa-2 **(b)** and SU.86.86 **(c)** tumors (50 mg/kg x 3 days/week x 2 weeks). Black bar represents administration of treatment. N=10 animals per treatment group. **(d)** Pimonidazole staining as biomarker of physical tumor hypoxia. Pimonidazole Hydrochloride was injected 2hr prior to tumor removal. Scale Bar = 300 μM **(e)** Carbonic Anhydrase IX (CAIX) staining as biomarker for tumor biological hypoxia. PPC analysis identified Hs766t and MiaPaCa-2 tumors as expressing significantly more CAIX than SU.86.86 tumors. **(f)** Histological staining of g-H2AX in PDAC tumors pre- and 48hr post-TH-302 treatment (50 mg/kg). **(g)** Nuclear stain intensity analysis of g-H2AX staining. g-H2AX significantly increased in Hs766t and MiaPaCa-2 Th-302 treated tumors. Data are presented as mean ± S.D of three tumor samples. A two-tailed Student’s t-test was used to determine significance. # p=0.06, ** p<0.01, ***, p<0.001. Scale Bar = 100 μM. See also Additional file 1: Figure S2.

Hs766t, MiaPaCa-2, and SU.86.86 pancreatic cancer cells were suspended in Matrigel Matrix (BD Biosciences, Franklin Lakes, NJ, USA) and phosphate-buffered saline (PBS) (1:1). Female SCID mice (6 weeks) (Harlan Inc., Indianapolis, IN, USA) were inoculated with 5 × 10^6^–10 × 10^6^ cells (matrigel/PBS) subcutaneously on the right flank to form xenograft tumors. Tumor volume was determined twice per week by caliper until the volume reached 300 mm^3^ upon which the mice were randomly placed into treatment groups (*N* = 10 per group). Treatment groups consisted of saline control, TH-302 (50 mg/kg (Figure 1) and 80 mg/kg) and pyruvate (1.15 mmol/kg) (Sigma-Aldrich, St. Louis, MO, USA) plus TH-302. TH-302 was administered IP five times per week for 2 weeks. Pyruvate was administered IV 30 min prior to TH-302 in combination studies. The tumor volumes were followed twice per week by caliper. Mice with tumors reaching 2,000 mm^3^ were removed from the study.

### Immunohistochemistry

Surgically removed pancreatic xenografts were formalin fixed and paraffin embedded (FFPE). Untreated xenograft tissue cross sections were stained for carbonic anhydrase IX (anti-rabbit AB15086; Abcam, Cambridge, MA, USA), lactate dehydrogenase V (anti-rabbit AB9002; Abcam), monocarboxylate transporter 1 (anti-rabbit SC-50324; Santa Cruz Biotechnology, Santa Cruz, CA, USA), and CD31 (anti-rabbit AB28364; Abcam). PDAC xenografts treated with TH-302 (50 mg/kg) for 48 h were formalin fixed, paraffin embedded, and stained for gamma-H2AX. Pimonidazole hydrochloride (60 mg/kg; Hypoxyprobe Inc.) was injected I.P. 1 h prior to tumor removal. Pimonidazole-positive tissue was detected using rabbit anti-sera against pimonidazole hydrochloride (2,627; Hypoxyprobe Inc.). A tissue microarray (TMA) containing FFPE human pancreatic adenocarcinoma specimen, acquired from Moffitt Cancer Center Total Cancer Care tissue database, was stained with antibodies against monocarboxylate transporter 1, monocarboxylate transporter 4 (anti-rabbit SC-376140; Santa Cruz Biotechnology), and pyruvate kinase isoform M2 (anti-mouse #3198; Cell Signaling Technology Inc., Danvers, MA, USA). The Ventana OmniMap anti-rabbit or anti-mouse secondary was used to detect primary antibodies. The detection system used was the Ventana ChromoMap Kit, and slides were then counterstained with hematoxylin. Histology stained slides were scanned using the Aperio ScanScope XT.

### Seahorse XF metabolic assays

*In vitro* metabolic analysis using the XF96 (Seahorse Bioscience, Chicopee, MA, USA) was previously described [18]. Briefly, real-time OC (pMole/min) and proton production rates (PPR, pMole/min) are determined by measuring oxygen and free protons in the media over a monolayer of cells. All collected data are normalized to protein using a standard BCA protein assay. Data are presented as the normalized mean ± standard deviation (S.D.)/mg protein.

### Mitochondrial stress test

ATP-linked OCR and mitochondrial reserve capacity were determined following the sequential injections of oligomycin (1 μM), carbonyl cyanide-p-trifluoromethoxyphenylhydrazone (FCCP) (1 μM), and rotenone plus antimycin A (1 μM). ATP-linked oxygen consumption is determined by the difference between basal OCR and OCR following addition of oligomycin. Mitochondrial reserve capacity is defined as the difference of basal OCR and OCR following the addition of FCCP.

### Glycolysis stress test

Glycolysis and glycolytic capacity were determined following sequential injections of D-glucose (2 g/L), oligomycin (1 μM), and 2-deoxyglucose (100 mM). Initially, the cells are glucose starved for 2 h prior to experiment start. Glycolysis was defined as PPR following the addition of D-glucose, and maximum glycolytic capacity was defined as PPR following the addition of oligomycin. PPR following treatment with 2-deoxyglucose is associated with non-glycolytic activity.

### Glucose and pyruvate uptake assay

Glucose (K676) and pyruvate (K609) uptake from culture media was measured using colometric from BioVision Inc. The PDAC cells were seeded in standard growth media (see “Cell culture” section) and allowed to adhere overnight. At *T* = 0, the cells were rinsed twice with PBS and provided DMEM supplemented with 5.5 mM D-glucose, 1 mM L-glutamine, 2 mM sodium pyruvate, and 0.2% FBS. Following 12 h of culture time, the media were collected, centrifuged to remove cell debris, and immediately tested for glucose and pyruvate following BioVision assay kit protocols and compared to starting media conditions. Absorbance was quantified at 570 nM. Data are presented as percent substrate uptake (mean ± S.D.) relative to *T* = 0 culture media concentrations.

### EPR imaging

A pulsed electroparamagnetic resonance (EPR) scanner with a parallel coil resonator tuned to 300 MHz was used for oxygen imaging. After the mouse was placed in the resonator, triarylmethyl EPR oxygen tracer OX063 (1.125 mmol/kg bolus followed by 0.12 mmol/min of continuous infusion) was administrated via tail veil cannula. Single-point imaging with a three-gradient set was used to generate T_2_^*^ map, i.e., EPR line width map, which linearly correlates with local concentration of oxygen and allows pixelwise estimation of pO_2_. In total, four pO_2_ maps were obtained for each mouse before, 10, 30, and 60 min after IV injection of pyruvate (1.15 mmol/kg, bolus). After EPR study, the mice were transferred to a 7T MRI scanner (Bruker Bio-Spin MRI GmbH) for anatomic imaging.

### Mathematical model

The previously developed mathematical model microPD [19], which couples the reaction-diffusion/convection equations with the fluid-structure interaction methods on spatially explicit tissue structure, has been calibrated to match the histology of MiaPaCa-2 mouse xenograft, the PK/PD properties of oxygen, and both inactive and active TH-302 (Additional file 1: Methods). The simulations were executed, visualized, and analyzed using a suite of in-house Matlab routines. Tissue histology was digitized using ImageJ software and in-house Matlab routines.

### Statistical analysis

A two-tailed unpaired Student’s *t*-test was used to determine statistical significance. The significance level was set at *p* < 0.05.

## Results

### Identifying baseline TH-302 sensitivity in human PDAC tumor models

Three human PDAC cell lines (Hs766t, MiaPaCa-2, and SU.86.86) were used to evaluate the effect of exogenous pyruvate on tumor hypoxia and TH-302 efficacy. Subcutaneous tumors were shown to be histologically (H&E) similar to pancreatic orthotopic tumors (Additional file 1: Figure S1). These were used to accommodate imaging modalities used to monitor pyruvate metabolism and tumor oxygenation, which cannot currently be made with orthotopic PDAC tumors. TH-302 monotherapy studies were carried out against each tumor type. The greatest anti-tumor effect occurred in Hs766t tumors. MiaPaCa-2 tumors had an intermediate response, and SU.86.86 tumors displayed no sensitivity to TH-302 (Figure 1a–c). As hypoxia is the main determinant for TH-302 activity, immunohistochemistry (IHC) detecting pimonidazole and carbonic anhydrase IX, respectively, markers for physical [20] and biological [21] hypoxia, confirmed the greatest hypoxia in Hs766t tumors (Figure 1d,e). Hs766t cells also carry a nonsense mutation in *FANCG* [22] rendering them deficient in DNA repair and hence sensitizing them to alkylating agents, such as Br-ifosphoramide, the effector agent in TH-302. To confirm sensitivity to such agents, all three lines were treated with DNA cross-linking agent, mitomycin C (MMC), under normoxic conditions. Hs766t cells exhibited extreme sensitivity to low nanomolar concentrations of MMC (IC_50_ = 2.8 nM) in comparison to MiaPaCa-2 (IC_50_ = 15.1 nM) and SU.86.86 (IC_50_ = 29.7 nM) cells which carry no detectable DNA repair defects (Additional file 1: Figure S2a). Negligible TH-302 activity in SU.86.86 may be attributed to a well-oxygenated tumor environment as evidence by low CA9 expression and reduced sensitivity to DNA cross-linking agents. *In vivo* tumor doubling times also correlated with TH-302 sensitivity, as faster growing tumors (Hs766t) were more hypoxic and sensitive to TH-302 than slower growing tumors (SU.86.86), which we interpret as a higher propensity to outgrow existing blood vessels (Additional file 1: Figure S2b). Quantification of vascular density based on CD31 staining supports this observation as Hs766t (*p* = 0.0526) and MiaPaCa-2 (*p* = 0.0189) tumors had less vascular density than SU.86.86 tumors (Additional file 1: Figure S3). Th-302 toxicity was validated by quantification of γ-H2AX, a DNA damage response reporter [5] (Additional file 1: Figure S2d). γ-H2AX increased in treated Hs766t (*p* = 0.004) and MiaPaCa-2 (*p* = 0.061) tumors with no change in SU.86.86 tumors (*p* = 0.669) (Figure 1f,g).

### Pyruvate stimulates oxygen consumption in glycolytic cells

**Figure 2:**
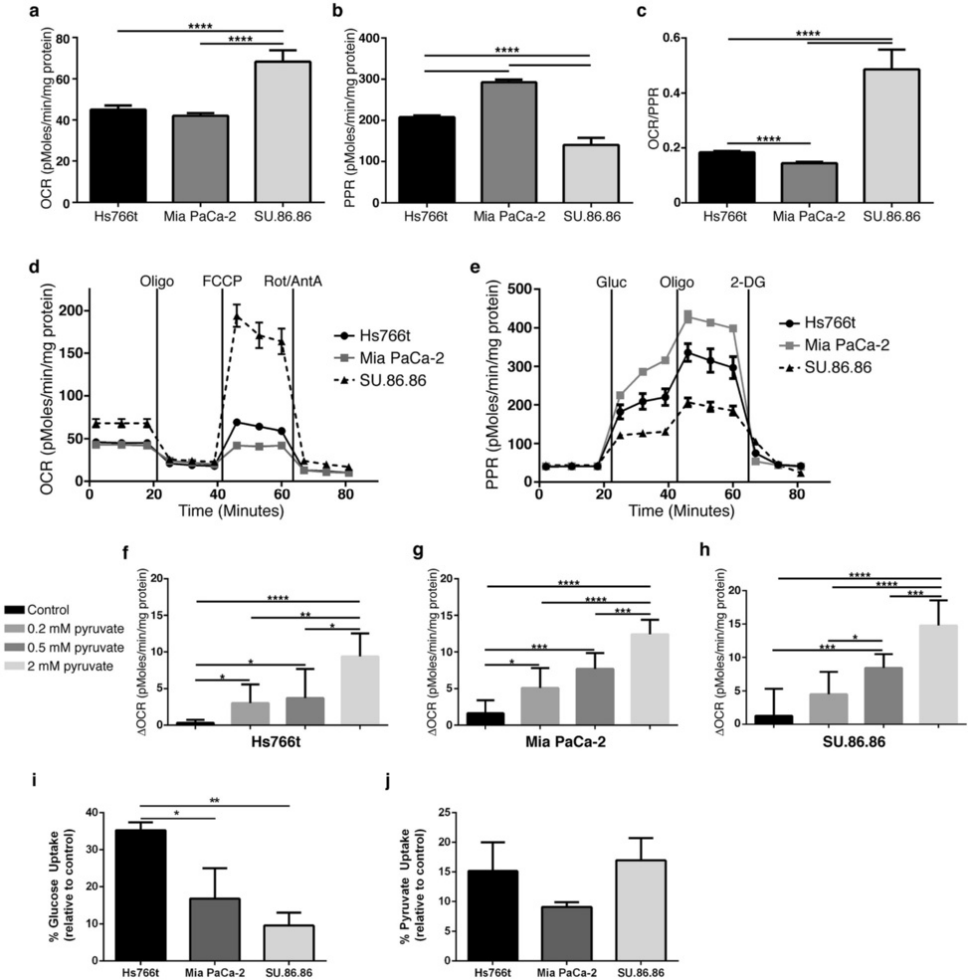
***In vitro*** **metabolic analysis of PDAC cell lines.** Basal oxygen consumption rates [OCR (pMoles/min/mg protein)] **(a)**, proton production rates [PPR (pMoles/min/mg protein)] **(b)** and OCR/PPR **(c)** measurements acquired using XF96 for all three PDAC cell lines. Data presented as mean ± S.D. **(d)** Mitochondrial bioenergetics profile indicates SU.86.86 cells as more oxidative than Hs766t and MiaPaCa-2 cells. Oligo = 1 μM oligomycin, FCCP = 1 μM FCCP and Rot/Ant = 1 μM Rotenone and 1 μM Antimycin A. **(e)** Glycolytic bioenergetics profile shows MiaPaCa-2 and Hs766t cells exhibit a glycolytic phenotype in comparison to SU.86.86 cells. Gluc = 12 mM D-Glucose, Oligo = 1 μM Oligomycin and 2-DG = 50 mM 2-Deoxyglucose. **(f-h)** ΔOCR following dose dependent administration of exogenous sodium pyruvate. Data are presented as mean ± S.D of three replicate studies. A two-tailed Student’s t-test was used to determine significance. * p<0.05, ** p<0.01, ***, p<0.001, **** p<0.0001. See also Additional file 1: Figures 3 and 4. (i) Percent glucose uptake and (j) percent pyruvate uptake presented relative to T=0 substrate concentrations. Data are presented as mean ± S.D. of three replicates.

The Seahorse XF96 was used to determine metabolic phenotypes (i.e., oxidative or glycolytic) for each cell line. The OCR of Hs766t (42 pMoles/min/mg) and MiaPaCa-2 (40 pMoles/min/mg) cells were significantly lower than that of SU.86.86 (65 pMoles/min/mg) (Figure 2a). Mitochondrial stress tests indicated that SU.86.86 cells had greater ATP-linked OCR and mitochondrial reserve capacity than Hs766t and MiaPaCa-2 cells, implying that the SU.86.86 cells are more oxidative (Figure 2d and Additional file 1: Figure S4a–c). Consistent with this interpretation, Hs766t and MiaPaCa-2 cells exhibited glycolytic phenotypes. Glucose-stimulated acid production (PPR) for Hs766t (179 pMoles/min/mg) and MiaPaCa-2 (275 pMoles/min/mg) cells were higher than that for SU.86.86 (87 pMoles/min/mg) (Figure 2b). ATP-linked glycolysis and glycolytic capacity were also greater in Hs766t and MiaPaCa-2 cells (Figure 2e and Additional file 1: Figure S4d–f). Glucose consumption was quantified and shown to be greatest in Hs766t and least in SU.86.86 cells partially supporting the Seahorse metabolic data (Figure 2i). These metabolic data suggest that Hs766t and MiaPaCa-2 cells are better suited to thrive under hypoxic conditions. Interestingly, SU.86.86 cells grew better under hypoxia than Hs766t and MiaPaCa-2 cells, even though the latter cell lines exhibited glycolytic phenotypes and formed more hypoxic tumors (Additional file 1: Figure S2c).

Pyruvate was administered to cells in increasing doses (0–2 mM) with expectations that respiration would increase. 2 mM pyruvate stimulated the largest OCR response (Figure 2f–h). Hs766t cells exhibited the largest relative increase and SU.86.86 cells the lowest, despite the fact that they had the largest oxidative reserve (Additional file 1: Figure S5e). No significant difference of pyruvate consumption was observed between cell lines, suggesting that intracellular pyruvate utilization may differ between cell lines (Figure 2j). PPR response to pyruvate was inversely related to OCR, presumably due to a reduction in net acid production (i.e., lactate derived from pyruvate rather than glucose), as well as a greater concentration of pyruvate entering the tricarboxylic acid cycle (TCA) cycle (Additional file 1: Figure S5a–d). Mitochondrial membrane potential increased following administration of 4 mM pyruvate in SU.86.86 cells as determined using tetramethylrhodamine ethyl ester (TMRE), confirming stimulation of the TCA cycle (Additional file 1: Figure S5f). These data indicate that cells with a glycolytic phenotype are more susceptible to pyruvate-stimulated OCR than oxidative SU.86.86 cells.

**Figure 3:**
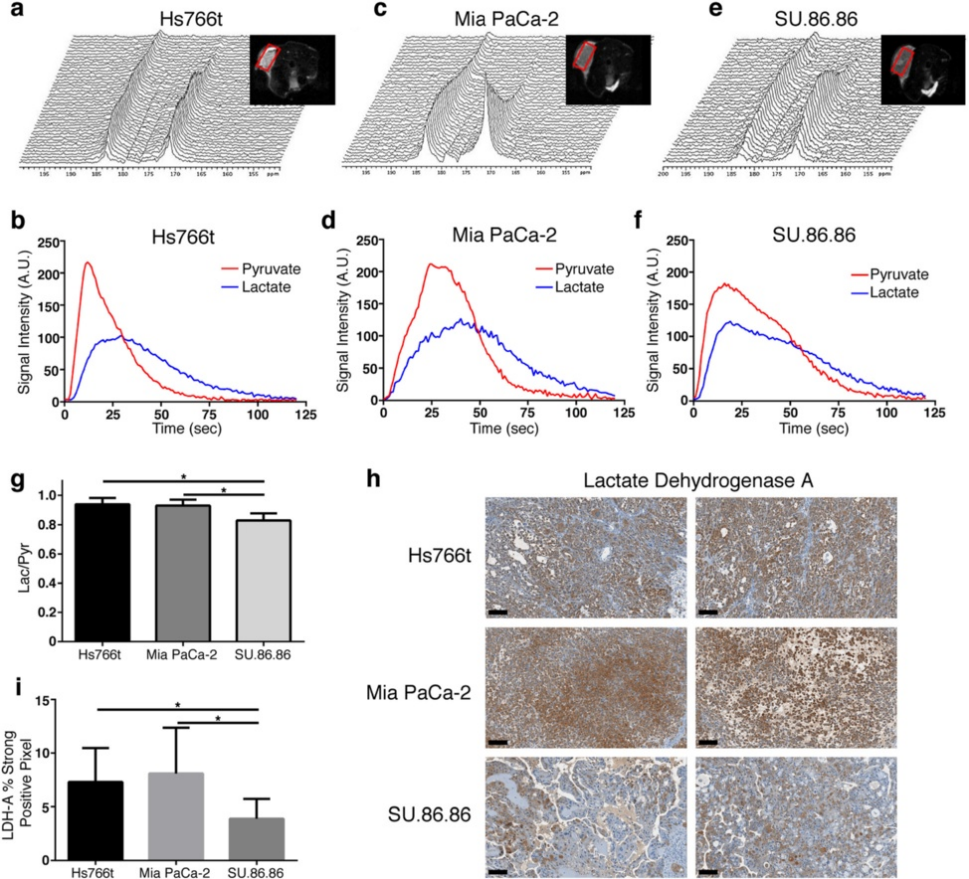
**Dynamic** ^**13**^ **C MR spectra following intravenous administration of pyruvate.** ^13^C MR spectra from Hs766t **(a)**, MiaPaCa-2 **(c)** and SU.86.86 **(e)** tumors. N=4 tumors per cell line. Pyruvate and lactate peaks are at 171 and 183 ppm respectively. Spectra were collected every 2 second. T2 weighted axial MR images containing slice ROI (red box) are inset. Tumor lactate and pyruvate peak intensities are shown with time for Hs766t **(b)**, MiaPaCa-2 (d) and SU.86.86 **(f)** tumors. **(g)** Lactate to pyruvate ratio (Lac/Pyr) for all three tumor types. (N=4 tumors for each group and presented as mean ± S.D.). **(h)** Lactate Dehydrogenase A (LDHA) histological staining and **(i)** PPC analysis in all three PDAC tumors. Expression of LDHA was significantly greater in Hs766t and MiaPaCa-2 tumors in comparison to SU.86.86. Data are presented as mean ± S.D of four biological replicates. A two-tailed Student’s t-test was used to determine significance. * p<0.05. Scale Bar = 100 μM.

We performed ^13^C magnetic resonance spectroscopy (MRS) of hyperpolarized [1-^13^C]pyruvate to validate the metabolic phenotypes *in vivo*. The pyruvate-to-lactate conversion in real-time is shown (Figure 3a–f), and the flux ratio (Lac/Pyr) was calculated from the area under the curve (AUC) for lactate and pyruvate versus time (*N* = 4). The Lac/Pyr flux ratio in Hs766t and MiaPaCa-2 tumors were significantly greater than that in SU.86.86 tumors (Figure 3g). Apparent rate constants *k*_P_ (Pyr/Lac) and *k*_L_ (Lac/Pyr) were calculated using modified Bloch equations [23]. The *k*_P_/*k*_L_ ratio was significantly higher in Hs766t (3.3 ± 0.5) and MiaPaCa-2 (3.4 ± 0.4) than SU.86.86 (2.7 ± 0.4) tumors. The Lac/Pyr flux and *k*_P_/*k*_L_ ratios corresponded with the glycolytic phenotype of Hs766t and MiaPaCa-2 observed *in vitro* (Figure 2e and Additional file 1: Figure S4d–f). Hs766t and MiaPaCa-2 tumors also histologically exhibited higher lactate dehydrogenase A protein expression than SU.86.86 (Figure 3h,i).

### Pyruvate transiently decreases tumor oxygenation

**Figure 4:**
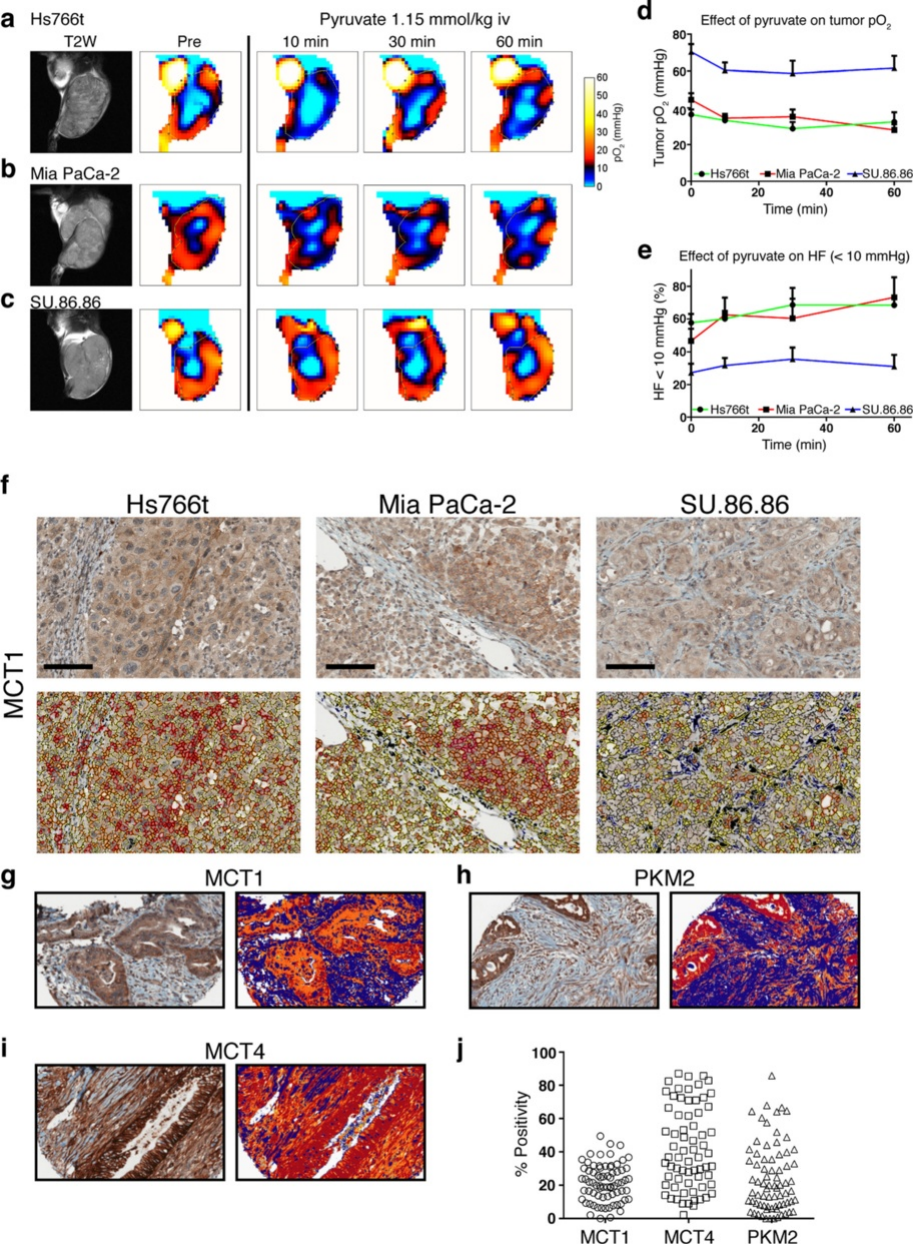
**EPR tumor oxygen imaging following administration of exogenous pyruvate. (a–c)** EPR oxygen imaging of subcutaneous Hs766t **(a)**, MiaPaCa-2 **(b)**, and SU.86.86 **(c)** tumors pre- and post (10–60 min) IV pyruvate administration. Representative T2 weighted anatomical MR imaging and pO_2_ maps are provided *N* = 4 biological replicates per tumor type. **(d)** Temporal changes of mean pO_2_ and **(e)** percent hypoxic fraction (<10 mmHg) of pyruvate-treated PDAC tumors. Data presented as mean ± S.D. Proposed histological predictive biomarkers for pyruvate sensitivity. **(f)** Histological staining of monocarboxylate transporter (*MCT*) 1 expression in PDAC subcutaneous tumors. Corresponding images of positive pixel membrane staining are included. Human PDAC tissue microarray stained for MCT1 **(g)**, MCT4 **(h)**, and pyruvate kinase isoform M2 **(i)**. **(j)** Distribution of percent positivity across well-differentiated human PDAC tissue cores. Scale bar = 100 μM. See also Additional file 1: Figure S5.

Electron paramagnetic resonance imaging (EPRI) was used to quantify changes in tumor pO_2_ following pyruvate administration [8]. Following anatomic MRI (left column (T2W)) and prior to EPRI, the triarylmethyl radical (TAM) OX063 tracer was injected intravenously (IV) and baseline tumor pO_2_ determined (Figure 4a–c). Hs766t and MiaPaCa-2 tumors were hypoxic with a mean pO_2_ of 9.1 ± 1.7 and 11.1 ± 2.2 mmHg, respectively (Figure 4d). Approximately 50% of the tumor volumes had a pO_2_ < 10 mmHg (Figure 4e), the pO_2_ at which TH-302 is activated [24]. The SU.86.86 tumors were less hypoxic with a mean tumor pO_2_ of 17.6 ± 2.6 mmHg. Previously published data show that the pO_2_ of normal mouse muscle is 20.8 ± 3.3 mmHg [25]. Only 28% of SU.86.86 tumor volumes had a pO_2_ < 10 mmHg. SU.86.86 consumed O_2_ at a higher rate *in vitro*, indicating that although there is no direct correlation between OCR and hypoxia in the steady state, it is likely modulated by the vascular oxygen supply. Hence, SU.86.86 tumors are better perfused, as observed by the higher AUC for pyruvate delivery (Figure 3f). Pyruvate (1.15 mMol/kg) was administered IV and tumor pO_2_ monitored and presented as mean tumor pO_2_ and hypoxic fraction (HF) of the entire tumor region. In SU.86.86 and Hs766t tumors, the greatest decrease in pO_2_ was observed 30 min post injection with pO_2_ recovering by 60 min (Figure 4a–d). In MiaPaCa-2 tumors, the pO_2_ continued to decline throughout the study with no indication of recovery. The HF (pixels <10 mmHg) significantly increased in both MiaPaCa-2 (22%) and Hs766t (12%) tumors. SU.86.86 baseline HF (28%) was considerably lower than the other two tumor types, increasing only 7%, 30 min after administering pyruvate, and returning to near baseline by 60 min (Figure 4e). Representative frequency histograms of tumor pO_2_ demonstrated a leftward shift after pyruvate administration in Hs766t and MiaPaCa-2 tumors with an increased percentage of pixels <10 mmHg (Figure 4f–h and Additional file 1: Figure S6a–f). The sensitivity of Hs766t and MiaPaCa-2 tumors to pyruvate *in vivo* corroborate the *in vitro* metabolic profiling studies (cf. Figure 2). Additionally, low tumor pO_2_ and high HF of Hs766t and MiaPaCa-2 tumors at baseline may contribute to their native sensitivity to TH-302 monotherapy (Figure 1).

To investigate underlying mechanisms for pyruvate sensitivity *in vivo*, untreated pancreatic tumors were stained for monocarboxylate transporter 1 (MCT-1), a high affinity Pyr/Lac membrane transporter (Km = 0.7 mM/3.5 mM) [26] (Figure 4f). Expression was higher in Hs766t and MiaPaCa-2 tumors, compared to SU.86.86. MCT-1 was quantified per cell, shown as a heat map in the lower panels of Figure 4f. Expression was heterogeneous with relatively small populations of high MCT-1 expressing cells (red) surrounded by low expressing cells (yellow or blue). To determine if this histological observation was clinically relevant, we stained a human PDAC tissue microarray for MCT-1 and additional glycolytic markers, PKM2 and MCT-4 [27] (Figure 4g–j). All markers exhibited strong staining patterns in poorly differentiated ductal epithelial cells, as well as in stromal fibroblasts, indicating that stromal cells have altered metabolic activity, similar to the cancer cells. Stroma exhibiting such staining patterns may also contribute to hypoxic exacerbation by increasing respiration in their compartment, and this is under active investigation.

### Effect of pyruvate on TH-302 efficacy

**Figure 5:**
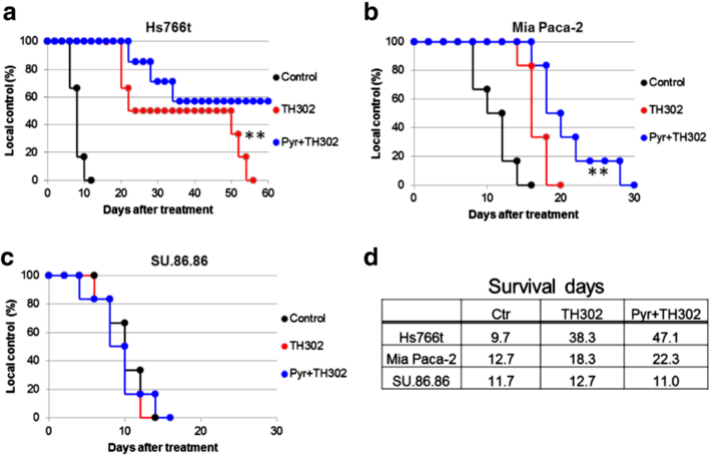
***In vivo*** **effect of pyruvate pretreatment on improving TH-302 efficacy.** Percent local tumor control of Hs766t **(a)**, MiaPaCa-2 **(b)**, and SU.86.86 **(c)** tumors were treated with saline, TH-302 alone (80 mg/kg × 5 days I.P.) or TH-302 following a 30 min pretreatment with exogenous pyruvate (1.15 mMol/kg pyruvate IV 30 min prior to 80 mg/kg TH-302 I.P. × 5 days). Response was measured as percentage of surviving animals as mice are removed from study when tumors reach 2,000 mm^3^. Pyruvate pretreatment significantly improved local control of Hs766t (*p* < 0.00225) and MiaPaCa-2 tumors with no measurable effect against SU.86.86. **(d)** Mean survival (days) of mice with pancreatic tumors treated with TH-302 and TH-302 in combination with pyruvate pre-treatment. *N* = 10 mice per treatment group. A two-tailed Student’s *t*-test was used to determine significance. **p < 0.01.

Since TH-302 is activated below 10 mmHg, we predicted that pyruvate pretreatment (30 min) would enhance TH-302 efficacy in Hs766t and MiaPaCa-2 tumors with little to no anticipated benefit in SU.86.86 tumors, as pyruvate had little effect on hypoxia in this model. PDAC tumors were established, and the mice were randomized into three treatment groups: 1) saline control, 2) TH-302 alone, and 3) pyruvate + TH-302 (pyruvate 30 min prior to TH-302). Data are presented as percent local control of tumor growth. SU.86.86 tumors remained resistant to TH-302 alone with no benefit in survival when combined with pyruvate (Figure 5c,d). Pyruvate had no effect on the initial Hs766t TH-302 tumor response, as they were already maximally responsive (Figure 1a). However, pyruvate pretreatment significantly improved overall Hs766t survival (Figure 5a,d) with 50% of the animals surviving beyond 54 days, at which time all animals in the other groups had expired. MiaPaCa-2 tumors exhibited the greatest decrease in tumor pO_2_ by exogenous pyruvate (Figure 4). These tumors received the greatest benefit from pyruvate pretreatment with a significant delay in tumor growth compared to both saline control and TH-302 monotherapy groups. Combining pyruvate with TH-302 increased the time to reach 2,000 mm^3^ compared to tumors treated with TH-302 monotherapy (*p* < 0.05) and overall survival significantly increased by 9.7 days (*p* < 0.005 in the pyruvate plus TH-302 treatment group compared to saline control) and by 4.7 days, compared to TH-302 monotherapy (log rank *p* < 0.05) (Figure 5b,d).

### MicroPD model

**Figure 6:**
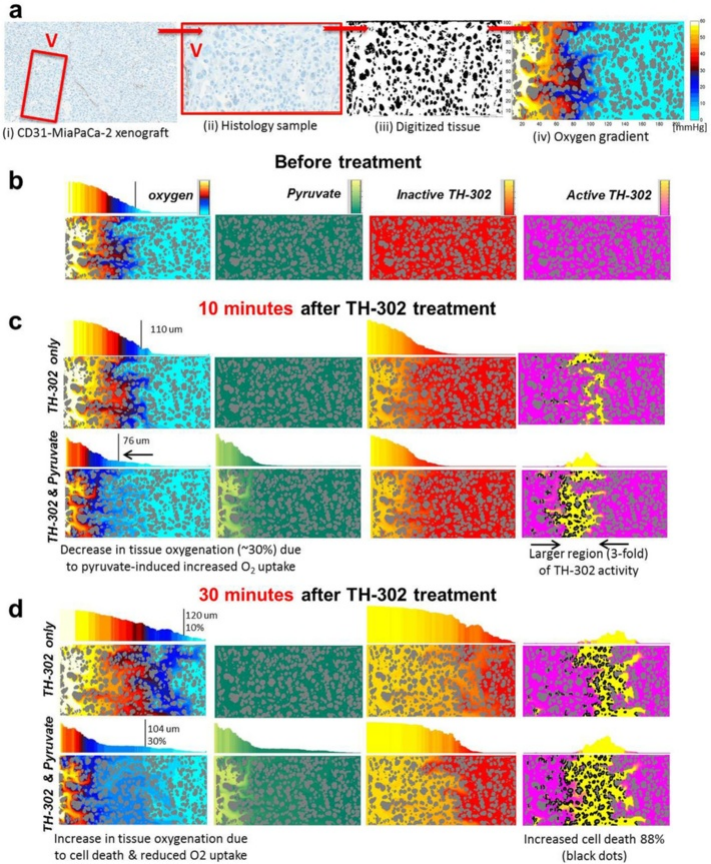
**Oxygen and active TH-302 distribution in simulated MiaPaCa-2 tissue extracellular space.** **(a)** A ROI of MiaPaCa-2 xenograft tissue stained with CD-31 (i) was selected (ii) for segmentation (iii), and used as a domain for in-silico simulations of the interstitial transport of oxygen (IV). **(b,c)** Temporal and spatial distributions of oxygen (1st column), pyruvate (2nd column), inactive TH-302 (3rd column) and active TH-302 (4th column). The bar graphs show averaged amounts of the compound across the tissue slice. The vertical lines in oxygen bar graphs show the border between the normoxic (left) and hypoxic (right) regions of the tissue. Black dots in the active TH-302 tissue graph represent dead cells. **(b)** Initial distribution of oxygen before the treatment is applied. Pyruvate, inactive and active TH-302 are all absent. **(c)** Distributions of chemical compounds 10 min after applying TH-302. In the bottom-row simulation pyruvate was applied 30 min before TH-302 (second column) which resulted in decreased pO2 due to increased oxygen uptake by the cells exposed to pyruvate (first column), and in expanded region of TH-302 activation (last column). **(d)** Distributions of chemical compounds 30 min after applying TH-302. In the bottom-row simulation, pyruvate was applied 30 min before TH-302 (second column). The region of TH-302 activation in two-fold larger (yellow region in the last column), and the number of death cells is 88% larger (black dots in the last column) when compared to simulation with TH-302 only.

The pharmacokinetics of combination therapies are complex. In order to attain quantitative understanding, a micro-scale spatially explicit reaction-diffusion/convection pharmacodynamics (microPD) model [19] parameterized with experimental measurements from MiaPaCa-2 tumors was developed to (refer to Additional file 1: Methods for model parameters) 1) explore TH-302 tissue penetration, 2) monitor TH-302 activation and diffusion of the released effector ± pyruvate, and 3) monitor tissue hypoxia ± pyruvate. For these analyses, tissue was digitized (Figure 6a) to spatially inform simulations (Figure 6b–d) to show diffusive distributions of oxygen, pyruvate, inactive TH-302, and active TH-302 at varying distances from vasculature (Figure 6b–d). The digitization contained individual cells, as well as interstitial parenchyma, that have the ability to consume oxygen, import pyruvate, and die when exposed to active TH-302. Baseline simulations showed that the hypoxia border (<10 mmHg) is, on average, 110 μm from the vasculature (Figure 6b). The measured diffusion distance of oxygen in tissue is between 100 and 200 μm [28]. With the addition of pyruvate (Figure 6c), cells closest to vasculature consumed more oxygen, reducing oxygen diffusion (hypoxia border = 76 μm) leading to a 30% hypoxia increase (Figure 2c). The leftward shift of the hypoxia border is paramount as the distance by which inactive TH-302 must diffuse prior to activation is shortened, increasing the region exposed to the effector threefold. TH-302 treatment in the presence of pyruvate increased cell death (black dots) by 88% when compared to TH-302 alone (Figure 6d). An unexpected result of these simulations was a mechanism for tissue re-oxygenation following combination treatment. A rightward shift (*T* = 30 post TH-302) of the hypoxia border from 76 to 104 μm occurs as oxygen consumption decreases as cell death increases. These simulations quantitatively illustrate how exogenous pyruvate may increase local hypoxia and thereby improve TH-302 efficacy.

## Discussion

Because hypoxic tumors are poorly prognostic and resistant to chemo- and radiotherapies, there has been intense interest in agents that will target hypoxic volumes. HAPs are a new class of anti-cancer therapeutics, and TH-302 is a leading candidate in clinical trials. The pharmacodynamic effects of these agents are complex and depend on vascular delivery, tissue penetration-to-hypoxic volumes, activation, and bystander killing. This is made even more complex by the short plasma half-lives of these agents. We thus contend that increased efficacy can be achieved through transient exacerbation of hypoxia in combination with advanced pharmacodynamics modeling. We have shown that exogenous pyruvate transiently exacerbates tumor hypoxia by increasing the rate of cellular OCR leading to improved TH-302 anti-tumor efficacy. This work was performed in three PDAC models: Hs766t, MiaPaCa-2, and SU.86.86 cells that have high, moderate, and low sensitivity to TH-302 monotherapy, respectively. Molecular and metabolic characterizations of these cells and tumors were consistent with their responses, as were their responses to pyruvate *in vitro* and *in vivo*. As predicted, pyruvate pretreatment caused a significant therapeutic benefit to the intermediately sensitive MiaPaCa-2 tumors exclusively. However, there were dramatic increases in survival for Hs766t tumors, which are highly sensitive to TH-302 monotherapy. Nonetheless, in contrast to the strong *in vitro* and *in vivo* effects of pyruvate on OCR and oxygen levels, the therapeutic benefits of combining pyruvate with HAP therapy, albeit significant, were only modest. Hence, we propose that additional optimization of timing and dose, using *in silico* models, can lead iteratively to improved therapeutic outcome of this novel combination therapy.

While the effect of exogenous pyruvate on metabolism has only recently been explored in the context of cancer [8,9], pyruvate has been thoroughly investigated and identified in neuronal models as the predominant mitochondrial substrate [10]. We therefore hypothesized that reduced pO_2_ in pancreatic tumors by exogenous pyruvate was a result of elevated OCR. However, this does not explain why exogenous pyruvate alone would stimulate oxygen consumption in cancer cells. MiaPaCa-2 and Hs766t are glycolytic, implying that the pyruvate flux should be substantial and not limiting for mitochondrial consumption. However, the OCRs of these two tumor types were the most strongly affected by pyruvate. One possible explanation is that PKM2, the enzyme that catalyzes the dephosphorylation of phosphoenolpyruvate to pyruvate, is located adjacent to the plasma membrane, whereas the mitochondria are peri-nuclear [29,30]. The rate of pyruvate production is regulated by PKM2 and expression is consistently observed to be upregulated in transformed cells exhibiting glycolytic phenotypes. Since enzymes of the lower half of glycolysis are localized near the plasma membrane, we hypothesize that PKM2 is closely associated spatially with these enzymes, making the fate of pyruvate a readily available substrate for lactate dehydrogenase, rather than transport into the mitochondria [29]. Hence, PKM2-produced pyruvate must traverse several microns of intracellular space, with diffusion gradients and intervening bioconversions, before it can be used to fuel respiration. With these distances, reaction-diffusion models predict that the local concentration of pyruvate at PKM2 would have to be at least 100 times higher than the Km for mitochondrial consumption for the mitochondrial pyruvate dehydrogenase to become saturated [31].

In contrast, exogenous pyruvate may have a different metabolic fate than pyruvate generated via glycolysis. Following uptake, the process of converting pyruvate to lactate continues as shown by hyperpolarized ^13^C pyruvate MR studies. However, since exogenous pyruvate can bypass PKM2, the available pyruvate concentration for transport into the mitochondria increases, stimulating respiration. Since pyruvate is delivered as a single bolus, exogenous pyruvate is quickly metabolized and the effect on respiration only transient; hence, the approximately 30-min decrease of tumor pO_2_ in our pancreatic tumor model (Figure 4).

Finally, chronic hypoxia has been shown to select for cells that are resistant to apoptosis [32,33]. Hence, there may be concern that exacerbating hypoxia may lead to enhanced progression or therapy resistance. In contrast to previous studies that induced hypoxia pharmacologically over many days [7], the current metabolic approach lasts <1 h, which is consistent with the blood half-life of TH-302. We contend that acute changes in pO_2_ will not lead to enhanced proliferation or resistance, but this will have to be investigated. Additionally, as pyruvate has also been shown to increase OCR in neurons, glia, and cardiac muscle [10-12], it is possible that this treatment may lead to hypoxia in normal tissues and increase TH-302 off-target effects. No increased toxicity of TH-302 was observed in our studies, and we reason that active maintenance of vessel tone in normal tissues, in contrast to tumors, exerts a protective effect by increasing blood flow to volumes with higher respiration. Nonetheless, further studies are warranted to investigate potential synergism between compounds that exacerbate tumor hypoxia and hypoxia-activated prodrugs.

## Conclusions

PDAC has one of the worst prognoses of all cancers due to limited efficacy of current therapies. The hypoxia-activated prodrug, TH-302, has shown promise clinically but advances are needed to improve its efficacy against late stage pancreatic cancer. This study supports the concept that temporarily increasing tumor hypoxia with pyruvate can improve the efficacy of HAPs, including TH-302, against PDAC and other cancer types in the clinic. While the effect of pyruvate on TH-302 activity *in vivo* was significant, future studies are necessary to optimize timing and dose, using *in silico* models, iteratively leading to improved therapeutic outcome of this novel combination therapy.

## Additional file

Additional file 1:
**Supplementary table and figures.** This document contains additional supplemental data and extended methods section for the mathematical model. In total, one table and five supplemental figures are included.
