# Supplementary material for: Pyruvate sensitizes pancreatic tumors to hypoxia-activated prodrug TH-302
Source: Cancer Metab. 2015 Jan 29;3:2. doi: 10.1186/s40170-014-0026-z (PMC4310189; doi:10.1186/s40170-014-0026-z)
Supplement: Additional file 1: — Supplementary table and figures. This document contains additional supplemental data and extended methods section for the mathematical model. In total, one table and five supplemental figures are included. [file 40170_2014_26_MOESM1_ESM.pdf]

Supplemental Data

Table 1.

Clinical Trial Registry Number	Cancer Type		Clinical Phase
**NCT00495144**	Advanced Solid Tumors	C	1/2
**NCT00742963**	+/- Doxorubicin in Advanced Soft Tissue Sarcoma	C	1/2
**NCT01440088**	+/- Doxorubicin in Unresectable or Metastatic Soft Tissue Sarcoma	A	3
**NCT01746979**	+ Gemcitabine in Untreated Unresecetable Pancreatic Adenocarcinoma	R	3
**NCT01522872**	+/- Bortezomib in Multiple Myeloma	R	1/2
**NCT01864538**	Advanced Melanoma	R	2
**NCT01381822**	+/- Sunitinib in RCC, GIST and Pancreatic Neuroendocrine Tumors	A	1/2
**NCT01497444**	+ Sorafenib in Unresectable Kidney or Liver Cancer	R	1/2
**NCT01833546**	Solid Tumors and Pancreatic Cancer (Japan)	R	1
**NCT01149915**	Advanced Leukemia	C	1
**NCT01721941**	+ Doxorubicin in Hepatocellular Carcinoma	N	1
**NCT01485042**	+ Pazopanib in Advanced Solid Tumors	A	1
**NCT02020226**	Cardiac Safety Study in Advanced Solid Tumors	R	1
**NCT01403610**	+/- Bevacizumab in High Grade Glioma	R	2
**NCT02093962**	+/- Pemetrexed in Non-squamous NSCLC	R	2
**NCT01144455**	+/- Gemcitabine in Previously Untreated Pancreatic Adenocarcinoma	A	2
**NCT00743379**	+ Gemcitabine, Docetaxel OR Pemetrexed in Advanced Solid Tumors	A	1/2
**NCT02047500**	+ Gemcitabine AND Nab-Paclitaxel in Previously Untreated Pancreatic Cancer	R	1

Table 1. TH-302 clinical trials. R = Recruiting; N = Not yet recruiting; C = Completed; A = Active no longer recruiting.

Supplemental Figure 1

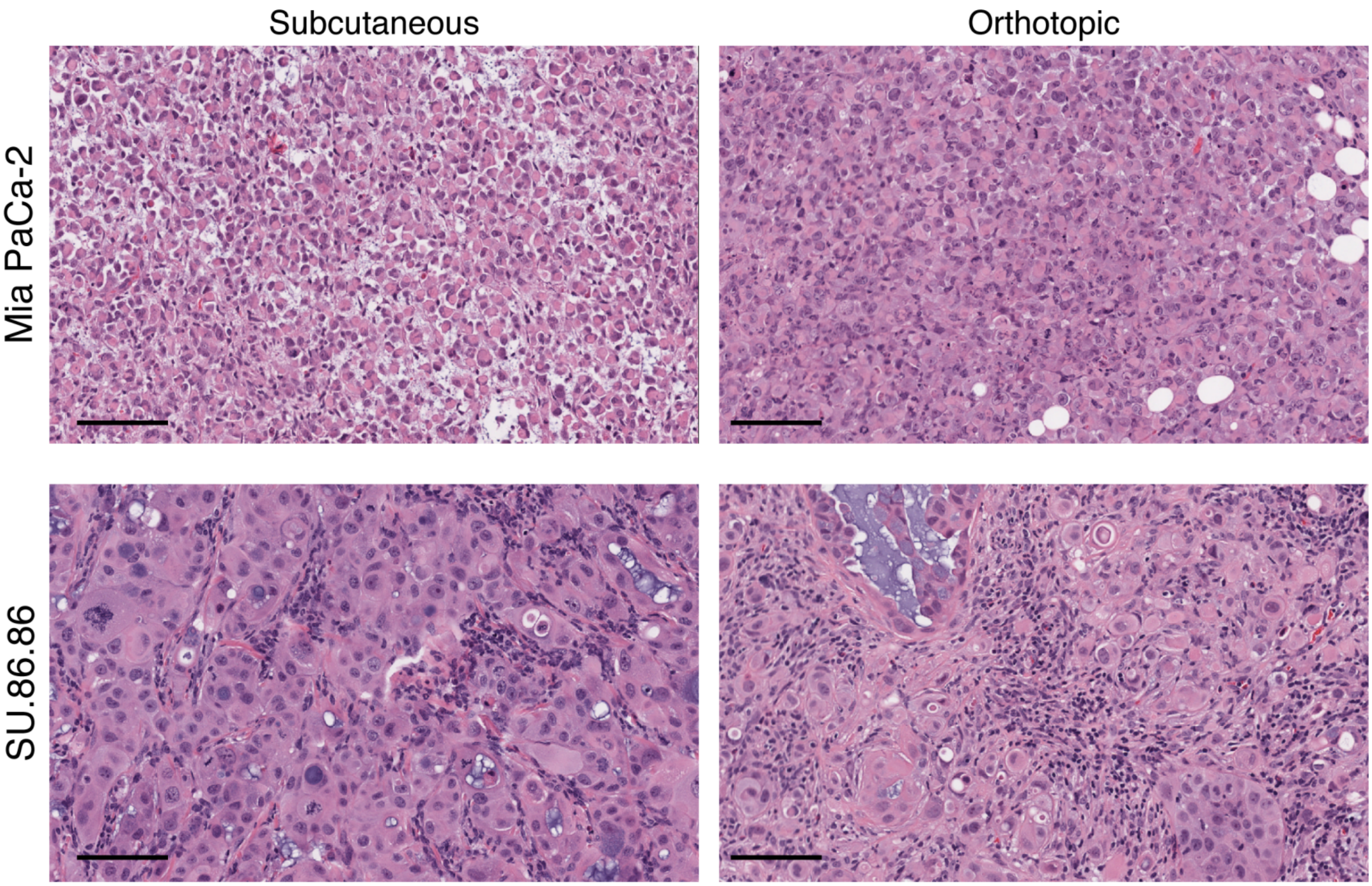

Supplemental Figure 1. Representative H&E images of Mia PaCa-2 and SU.86.86 orthotopic and subcutaneous tumors. Scale bar = 200 µM.

Supplemental Figure 2

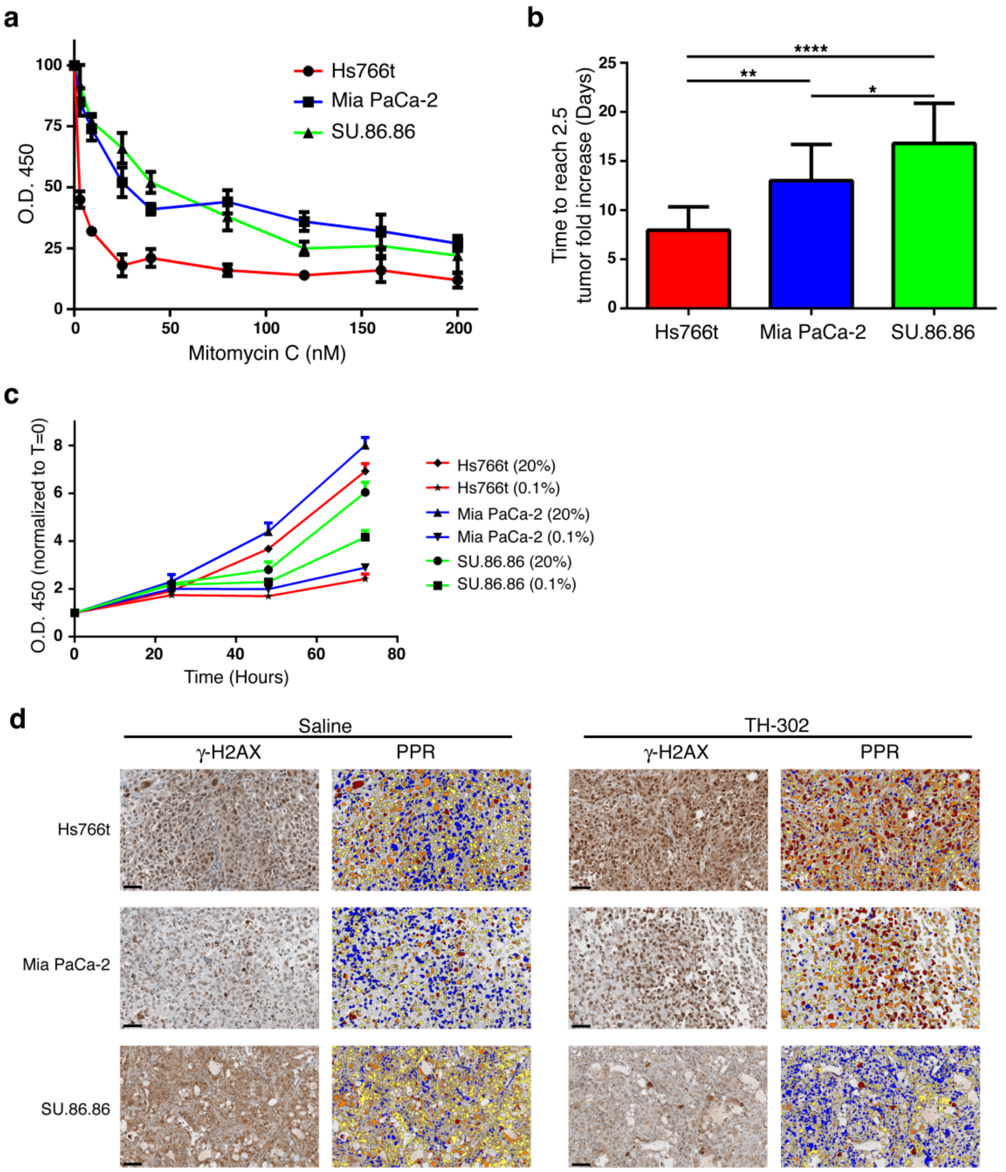

Supplemental Figure 2. (**a**) Viability of PDAC cell lines following dose-dependent Mitomycin C treatment. Data normalized to untreated controls. Data presented as mean ± S.D. **(b)** In-vivo tumor doubling times of subcutaneous PDAC tumors. All data presented as mean ± S.D. **(c)** In-vitro growth curves of PDAC cell lines grown under normoxic (20% O2) and hypoxic (0.2% O2) conditions. Data presented as mean ± S.D. **(d)** Representative pixel analysis routinely applied throughout this study to quantify staining intensity on a per pixel basis. Pixels are binned into four groups based on staining intensity; negative pixel (blue), weak positive pixel (yellow), moderate pixel (orange) and strong positive pixel (red). This analysis is of γ-H2AX pre- and post-TH-302 therapy in all three PDAC

subcutaneous tumors. γ-H2AX staining significantly increased in Hs766t and Mia PaCa-2 samples. * $p<0.05$, ** $p<0.01$, **** $p<0.0001$. Scale Bar = 100 μM.

Supplemntal Figure 3

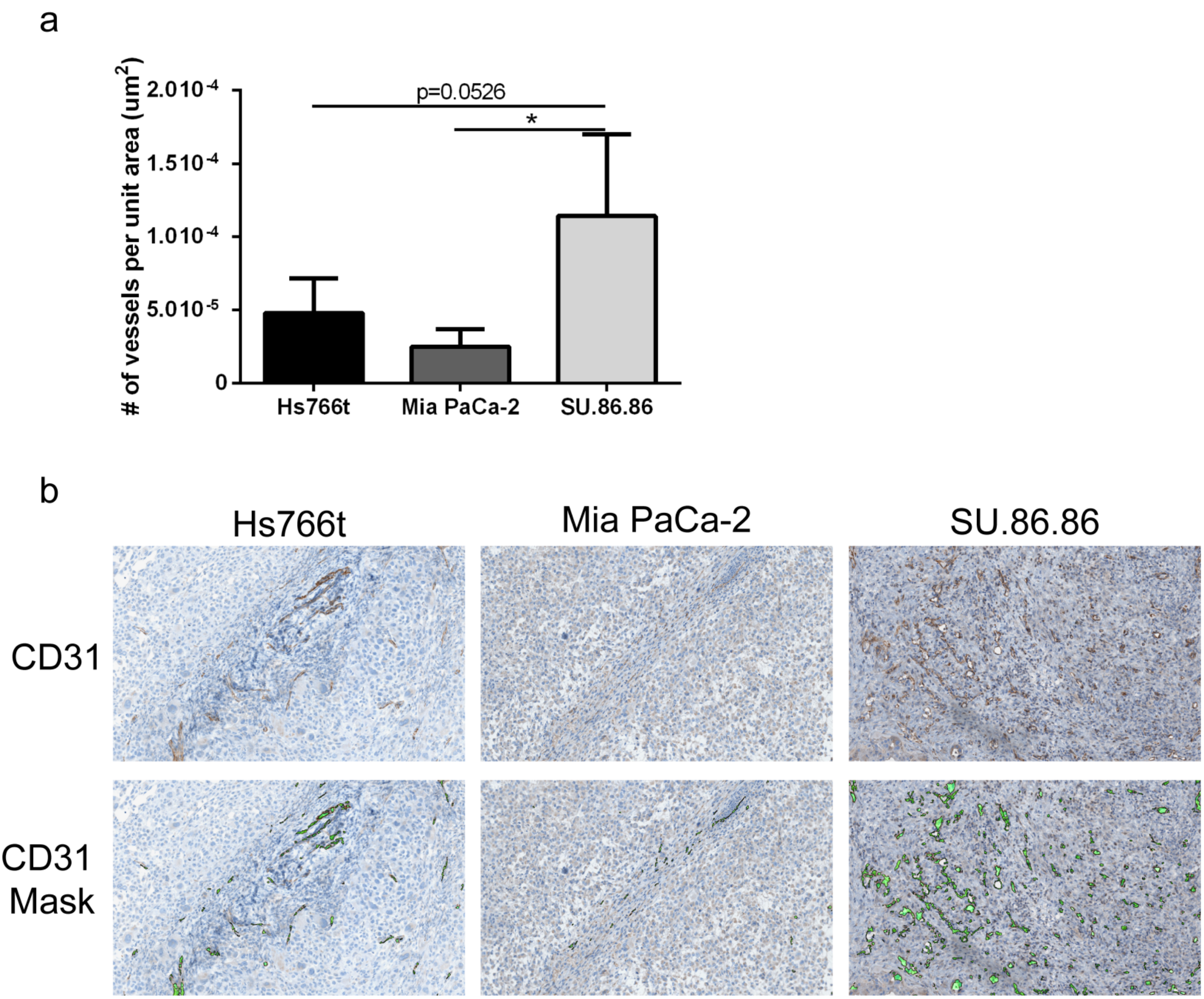

Supplemental Figure 3. (a) Vessel density of each tumor type based on CD31 IHC staining. Data are presented as number of vessels per um2 (average ± S.D.). * $p< 0.05$. **(b)** Representative CD31 staining for each tumor type. CD31 mask images are examples of algorithmically determined vasculature used to quantify vessel density.

Supplemental Figure 4

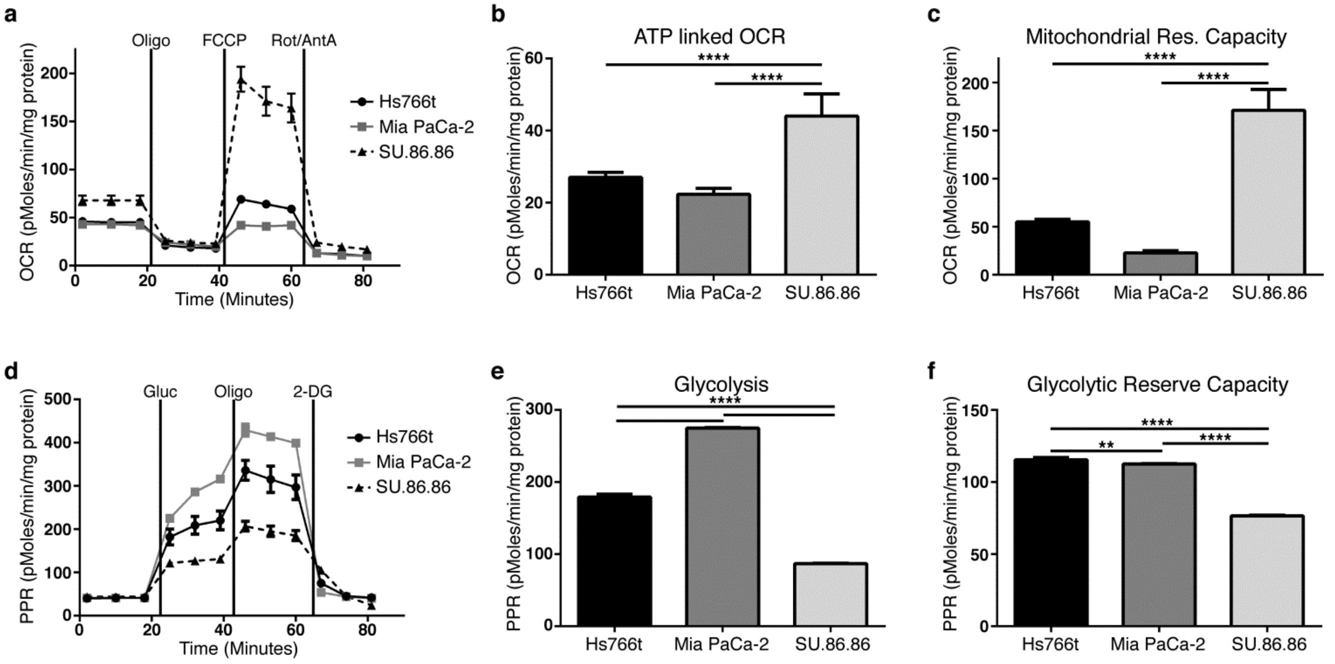

Supplemental Figure 4 (a) Mitochondrial stress test profiles for each PDAC cell line. **(b)** Mitochondrial ATP linked oxygen consumption and **(c)** mitochondrial reserve capacity. SU.86.86 cells have significantly more oxygen ATP linked oxygen consumption and reserve capacity. **(d)** Glycolytic stress test profiles for each PDAC cell line. **(e)** Glycolytic acidification and **(f)** glycolytic reserve capacity. Hs766t and Mia PaCa-2 cells exhibit a more glycolytic bioenergetics profile than SU.86.86. All data are presented as mean ± S.D. ** p<0.01, **** p<0.0001.

Supplemental Figure 5

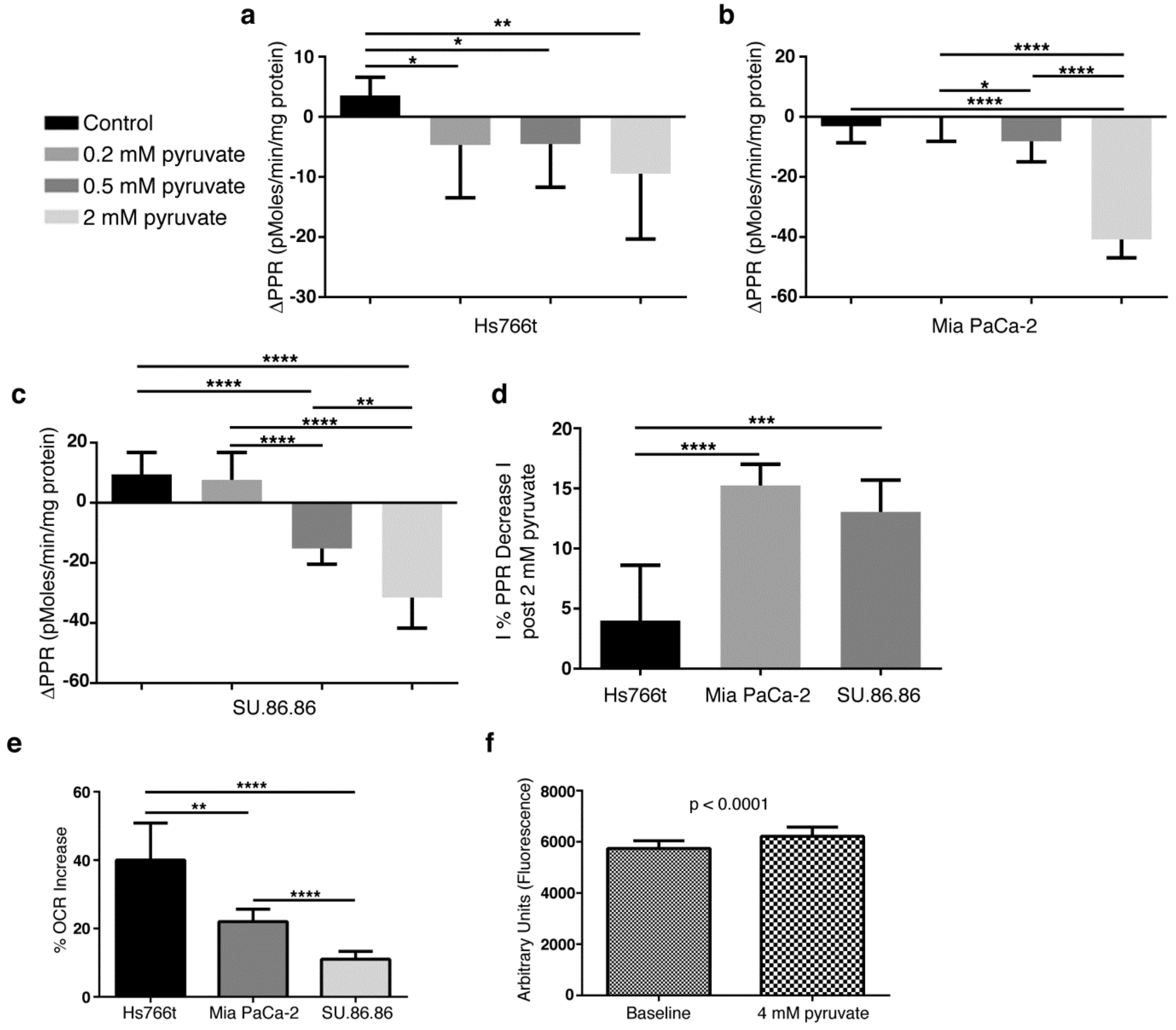

Supplemental Figure 5. Proton production rate (PPR) measurements following administration of pyruvate in a dose dependent manner in **(a)** Hs766t, **(b)** Mia PaCa-2 and **(d)** SU.86.86 cells. **(d)** Absolute percent change following 2 mM pyruvate administration. **(e)** Percent OCR change above basal OCR. Hs766t and MiaPaCa-2 %OCR increase was significantly greater than SU.86.86 cells. **(f)** Mitochondrial membrane potential measured in control and sodium pyruvate (4 mM) treated SU.86.86 cells using TMRE. Data are presented as mean ± S.D. * $p<0.05$, ** $p<0.01$, *** $p<0.001$, **** $p<0.0001$.

Supplemental Figure 6

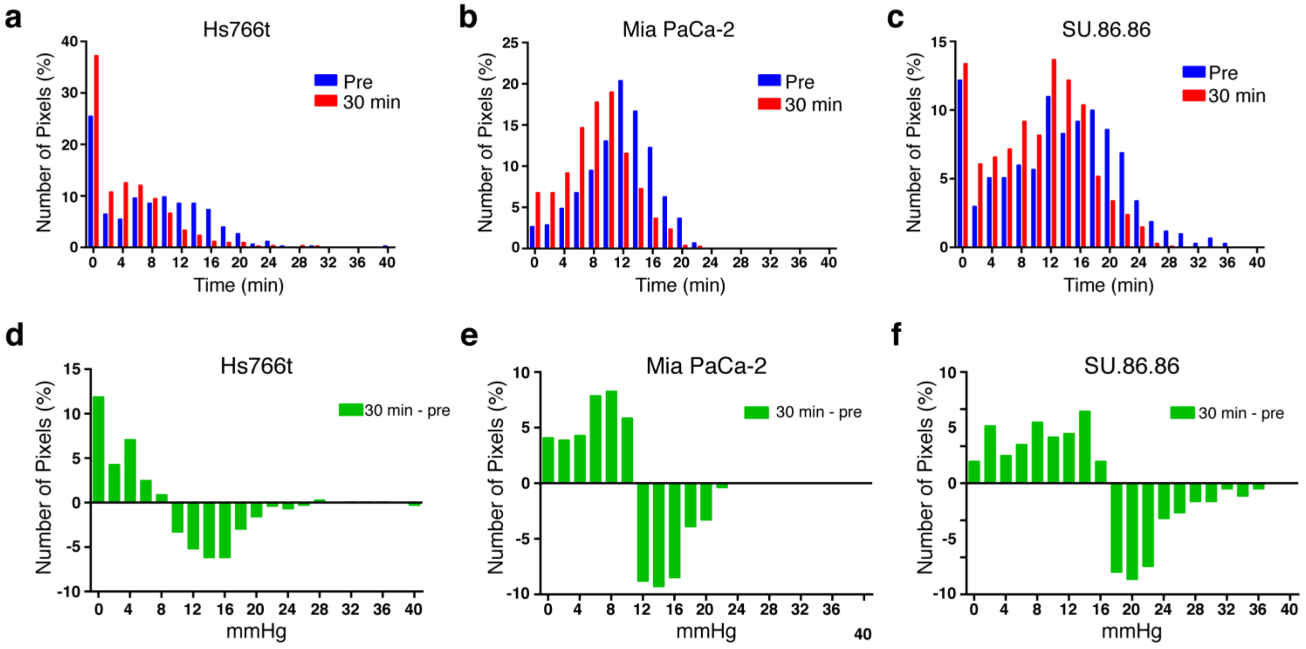

Supplemental Figure 6. (a-c) Tumor oxygenation (30 min post pyruvate minus baseline) displayed as percent pixel per pO2 (mmHg). **(d-f)** Frequency histogram of tumor pO_2 pre (Blue) and 30 min post (Red) pyruvate administration.

Supplemental Experimental Procedures

Immnuohistochemical Image Analysis: Pixel analysis of IHC tissue samples was previously described(2). Briefly, Aperio PositivePixelCount ® v9.0 algorithm optimized for whole cell, nuclear and membrane staining were used to determine staining intensity for slides stained for Pimonidazole Hydrochloride, Carbonic Anhydrase IX, gamma-H2AX, lactate dehydrogenase V, monocarboxylate transporter 1, monocarboxylate transporter 4 and pyruvate kinase isoform M2. The following thresholds [HueValue=0.1; HueWidth=0.5; Iwp(High)=220; Iwp(Low)/Ip (High)=175; Ip(Low)/Isp (High)=100; Isp(Low)=0; Inp(High)=-1] were used to segment negative (0), low (1+), moderate (2+) and strong positive (3+) pixels by mean stain density (0-255 dynamic range). Quantified data are presented as mean % strong positive pixel (total strong positive/(low + moderate + strong pixel) ± S.D.

Vascular density of CD31 stained PDAC FFPE samples was algorithmically determined using the Aperio vasculature algorithm with the following settings (Lumen and closed vessels including incomplete vessels with filtering =2; low = 160; high = 210; with stain components .27, .57 and.78 [RGB]. This algorithm was used to export the quantified values for number of vasculature per unit area (μm^2).

Proliferation Assay and Mitomycin C toxicity studies: Cells were seeded in a 96 well format and grown in either normoxic (20%) or hypoxic (0.1%) conditions (Xvivo Closed Incubation System, BioSpherix Ltd., Lacona, NY). Samples were collected every 24 hours for 72 hours to determine growth rates under both conditions. Cell growth was a measurement of biomass increase by crystal violet staining (Gillies et al., 1986). Mitomycin C (Tocris Biosciences, Minneapolis, MN) toxicity was determined in a dose dependent manner following 72 hours of treatment. Cell toxicity was a measurement of biomass increase by crystal violet staining. Data were presented as % untreated control. Graphpad prism was used to determine Mitomycin C LD50 for each PDAC cell line.

Hyperpolarization of [1-13C] pyruvate: Subcutaneous PDAC xenograft tumors were grown on the right flank in SCID mice. When tumor volumes reached 500 mm^3, a jugular catheter was surgically implanted to facilitate [1-13C] injections. For the MRI/MRS studies, mice were induced with isofluorane in a plastic anesthesia chamber with scavenging. Once unconscious, they were placed in a mouse-specific holder within the MRI coil, outfitted with a mouse-specific nose cone inhalant anesthesia and scavenging system for imaging. This system also contained a pad for respiration monitoring, an endorectal fiber optic temperature monitoring probe, and a heated pad for maintaining core body temperature at 37° C. Samples of [1-^{13}C] pyruvic acid (Isotech, Sigma Aldrich, St. Louis, MO, USA) containing 15mM of the OX063 trityl radical (Oxford Instruments) were hyperpolarized using the prototype Hypersense DNP polarizer (Oxford Instruments, UK) for an hour. Hyperpolarized pyruvic acid was rapidly dissolved in 4mL solution containing Tris/ETDA, NaCl and NaOH to achieve a final pH of 7.8 and injected into mice.

Following the injection of hyperpolarized pyruvate, single-transient ^{13}C spectra were acquired every 2 s over a period of 150 s using 5° flip-angle, T_R = 1s, 40 k data points and a spectral width of 20 kHz from a 5mm thick slice across the tumor. Peak area integration was performed and the intensity of the hyperpolarized pyruvate and lactate peak was plotted with time. The lactate-to-pyruvate flux ratio (Lac/Pyr) was calculated from the area under the curve (as regarded a model-free approach). All data are reported as the mean ± S.D. Statistical significance was determined using Student's t-test with $p<0.05$ considered as significant. Anatomical reference images to locate the xenograft tumor's slice were acquired with T_2-weighted FSE (fast spin echo) multislice (T_R = 4 s, TE = 60 ms, echo train length = 8, matrix 128 x 128, slice thickness = 1 mm, 15 slices) as shown in Figure A,C and E for Hs766t, Mia PaCa-2 and SU.86.86 tumors respectively.

Mathematical Model Supplement: The mathematical model, microPD (3) operates on the mesoscopic scale (cell and tissue) and couples the explicitly defined tissue morphology, mechanical properties of tumor interstitium and dynamical transport of compounds through the vasculature and the extracellular space filled with the interstitial fluid. microPD is a multiscale spatially explicit

reaction-diffusion-convection model based on the method of regularized Stokeslets (4) that captures interactions between the interstitial fluid, tumor cells and gradients of metabolites and drugs.

***Model equations*:** The microPD model is defined on a patch of digitized tissue containing vascular architecture and individually modeled tumor and stromal cells. We assume that the void space between the cells is filled with the interstitial fluid, which is modeled using the incompressible Stokes equations, **Eq.(1)**, where m is the fluid viscosity, *p* is the pressure, *u* is the fluid velocity, and *f* is the force applied at the capillaries and at the cells boundaries to create a physiologically relevant flow of the interstitial fluid from the vessel and around the cells. The mathematical framework of this model is based on the method of regularized Stokeslets (4), a classic fluid-structure interactions method, that can be solved explicitly upon the right choice of the regularization function, **Eq(2)**. We assume that the interstitial fluid field will contribute to the advective transport of various chemical compounds penetrating the tissue, **Eqs.(3-6)**. All chemical compounds (oxygen, TH-302, pyruvate) are subject to similar transport modalities: they are supplied from the blood system and are moved through the extracellular space due to their diffusivity and advective transport with the interstitial fluid flow. Certain compounds are absorbed by the cells (oxygen, active TH-302, pyruvate); others need to be activated (TH-302 pro-drug); some will decay over time (TH-302, pyruvate). The typical kinetics of these compounds are listed in **Eqs.(3-6)**.

$$\mu\Delta \mathbf{u}(\mathbf{x}) = \nabla p(\mathbf{x}) - \mathbf{f}(\mathbf{x}) \quad \text{and} \quad \nabla\cdot\mathbf{u}(\mathbf{x}) = 0 \tag{1}$$

$$\text{where} \quad \mathbf{f}(\mathbf{x}) = \mathbf{f}_0\, \phi_\varepsilon(\mathbf{x}-\mathbf{x}_0), \quad \text{and} \quad \phi_\varepsilon(\mathbf{x}) = \frac{2\varepsilon^4}{\pi(\|\mathbf{x}\|^2+\varepsilon^2)} \tag{2}$$

$$\frac{\partial c(\mathbf{x},t)}{\partial t} = \underbrace{\mathcal{D}_c\Delta c(\mathbf{x},t)}_{diffusion} - \underbrace{\mathbf{u}(\mathbf{x},t)\cdot\nabla c(\mathbf{x},c)}_{advection} - \underbrace{\frac{\kappa_m c(\mathbf{x},t)}{\kappa_n + c(\mathbf{x},t)}\chi(\Omega_\Gamma)}_{cellular\ \ uptake} \tag{3}$$

$$\frac{\partial \eta_i(\mathbf{x},t)}{\partial t} = \underbrace{\mathcal{D}_{\eta_i}\Delta \eta_i(\mathbf{x},t)}_{diffusion} - \underbrace{\mathbf{u}(\mathbf{x},t)\cdot\nabla \eta_i(\mathbf{x},c)}_{advection} - \underbrace{\xi(c(\mathbf{x},t))\eta_i(\mathbf{x},t)}_{activation} - \underbrace{\omega_i\eta_i(\mathbf{x},t)}_{decay} \tag{4}$$

$$\frac{\partial \eta_a(\mathbf{x},t)}{\partial t} = \underbrace{\mathcal{D}_{\eta_a}\Delta \eta_a(\mathbf{x},t)}_{diffusion} - \underbrace{\mathbf{u}(\mathbf{x},t)\cdot\nabla \eta_a(\mathbf{x},c)}_{advection} + \underbrace{\xi(c(\mathbf{x},t))\eta_i(\mathbf{x},t)}_{activation} - \underbrace{\alpha\eta_a(\mathbf{x},t)\chi(\Omega_\Gamma)}_{cellular\ \ uptake} \tag{5}$$

$$\frac{\partial \gamma_*(\mathbf{x},t)}{\partial t} = \underbrace{\mathcal{D}_{\gamma_*}\Delta \gamma_*(\mathbf{x},t)}_{diffusion} - \underbrace{\mathbf{u}(\mathbf{x},t)\cdot\nabla \gamma_*(\mathbf{x},c)}_{advection} - \underbrace{\beta\gamma_*(\mathbf{x},t)\chi(\Omega_\Gamma)}_{cellular\ \ uptake} - \underbrace{\omega_{\gamma_*}\gamma_*(\mathbf{x},t)}_{decay} \tag{6}$$

$$\text{initial conditions:} \quad \mathbf{u}(\mathbf{x},t_0) = \mathbf{u_0}(\mathbf{x}), \;\; c(\mathbf{x},t_0) = c_0(\mathbf{x}), \;\; \eta_i(\mathbf{x},t_0) = \gamma_i(\mathbf{x},t_0) = 0 \tag{7}$$

$$\text{vessel boundary conditions:} \quad \mathbf{u}(\mathbf{x_0},t) = \mathbf{u^0}, \; c(\mathbf{x_0},t) = c^0, \; \eta_i(\mathbf{x_0},t) = \eta_i^0, \; \gamma_*(\mathbf{x_0},t) = \gamma_*^0 \tag{8}$$

Model equations. u is the velocity of the interstitial fluid of viscosity μ, that is governed by the pressure differences *p* and the external forces ***f***; *c* is the oxygen concentration, η_i is an inactive TH-302 prodrug concentration, and η_a is an active TH-302, γ* represent concentration of pyruvate.

Model calibration: (1) Digitization of tissue histology. A slice of tumor tissue from Mia PaCa-2 xenograft was stained with CD-31 to localize tumor vasculature and scanned. From the obtained digital image a small patch of tissue was selected in such a way to contain a vessel along the edge. The image was converted to monochrome scale and individual cell shapes were captured using the ImageJ (NIH) software. Coordinates of all points forming the tumor cells were determined by in-house Matlab routines and were used to define the computational domain for further model simulations. (2) Parameters of the interstitial fluid flow were fitted to the values known from literature (see Model parameter table). (3) Parameters of oxygen kinetics were fitted to the values known from literature (see Model parameter table). (4) Parameters of TH-302 kinetics were fitted to values published by the Threshold Pharmaceuticals, Inc. group (see Model parameter table). (5) Parameters of pyruvate kinetics were determined experimentally by our group. (6) Parameters of Mia PaCa-2 cells were determined experimentally by our group. All parameter values are listed in the table below.

Mode Parameter	symbol	range/units	source
Interstitial fluid:			
viscosity	μ	1-10cP	
flux	u^0	0.1-2mm/s	(Chary & Jain, 1989)
Oxygen:			
input concentration	c^0	180μM	calculated from the oxygen level in the blood
diffusion	D_c	$0.6\text{-}2\times10^{-5}cm^2/s$	(Chresand et al., 1988)
uptake by cells	k_m, k_n		fitted from cell culture of Mia PaCa-2 using Seahorse XF
Inactive TH-302:			
input concentration	η_i^0	1-100mg/ml	calculated from TH-302 concentration in the plasma (Jung et al., 2012)
diffusion	$D\eta_i$	$\sim10^{-6}cm^2/s$	fitted from TH-302 molecular mass [Provided by Threshold]
half-life	ω_i	8min-0.8hr	(Jung et al., 2012)
activation ratio	ξ		fitted from the activation curves in 0-21% O_2 (Duan et al., 2008)
activation O_2 level	c_{thr}	0-0.6%O_2	(Duan et al., 2008)
Active TH-302:			
diffusion	$D\eta_a$	$\sim10^{-6}cm^2/s$	assumed equal to diffusion of inactive TH-302
half-life	ω_a	8min-0.8hr	assumed equal to inactive TH-302
uptake	α		determined in cell culture for Mia PaCa-2 and tested in silico
lethal level	η_{thr}		determined in cell cultures for Mia PaCa-2
Pyruvate:			
input concentration	γ_0	0.2-2 mM	fitted from pyruvate concentration in the plasma
diffusion	$D\gamma$	$\sim10^{-5}cm^2/s$	fitted from molecular mass
half-life	$\omega\gamma$		determined experimentally for Mia PaCa-2
uptake	β		determined in cell culture for Mia PaCa-2 and tested in silico
sensitizing level	γ_{thr}		determined experimentally for Mia PaCa-2
Tissue:			
architecture & vasculature			determined from ex vivo histology of Mia PaCa-2 tumors
diffusivity			determined using stirred bath technology (Chresand et al., 1988)

Model outputs: (1) Tissue metabolic homeostasis. As an initial step, we simulated tissue oxygenation in the absence of any treatment (**Fig. 6b**). In this case the balance is reached between oxygen supplied to the tissue from a vessel (located along the edge of the computational domain) and oxygen uptake by all tumor cells inside the tissue, and this steady state persists over the time considered in the simulation (oxygen level graphs and oxygen distribution snapshots in **Fig. 6b** taken over 60 minutes of simulated time). Since the cellular structure of the Mia PaCa-2 tumor tissue is heterogeneous, the steady-state pattern of oxygen penetration is irregular; as is the border between the normoxic and hypoxic regions of the tissue (cyan color indicates the hypoxic areas, where oxygen level is below 10mmHg). However, when the average value of O2 concentration is taken over the tissue width (bar-graphs in the top parts of **Fig. 6b**), the hypoxic border is around 110 μm from the vasculature (vertical line in Supplemental Figure 6B top row, left column, top figure). (2) Simulation of TH-302 monotherapy. TH-302 is supplied from the vessel in its inactive form, and becomes activated

upon reaching the hypoxic region of the tissue (snapshots in **Fig. 6c, third column**: distribution of inactive TH-302, last column: distribution of active TH-302). High concentration of the inactive TH-302 (yellow) coincides with the areas of high levels of oxygen (white, yellow and read), whereas the distribution of active TH-302 (yellow) coincides with the tissue hypoxic areas (cyan). The spatial patterns of high concentrations of both an inactive and activated HTH-302 are complementary. The active TH-302 when absorbed by the tumor cells leads to their death (black cell boundaries in **Fig, 6c,d, last column**). Over the time of 30 minutes after applying the TH-302, the area of TH-302 activity and the extent of death cells increased significantly (**Fig. 6d top row**). The level of oxygen and the extent to inactive TH-302 penetration increases in the middle part of the tissue due to fact that dead cells stop consuming the oxygen, and thus the normoxia-hypoxia border is shifted to the right about 10 microns (**Fig. 6d top row, first column** representing the averaged oxygen concentration in the tissue region of 80-120 microns from the vessel). (3) Simulation of pyruvate in combination with TH-302. When pyruvate is supplied from the vessel (spatial distribution shown in **Fig. 6c,d, bottom rows**), the near-by tumor cells are exposed to high concentration of pyruvate, which effects in an increased consumption of oxygen. As a result, oxygen penetration into the tissue is diminished by about 30%, and the location of the normoxic-hypoxic border is shifted to 76 microns from the vessel (Supplementary Figure 6C bottom row, first column). When TH-302 is applied 30 minutes later the increased hypoxic region results in a larger area of TH-302 activation (3-fold increased shown in **Fig. 6c bottom row, last column**). The area of inactive TH-302 penetration is smaller and coincides with the region of oxygen penetration. After next 30 minutes, the amount of dead cells was increased by 88% (**Fig. 6d bottom row, last column**). Again, since the dead cells stop consuming oxygen, the normoxia-hypoxia border is shifted to the right and reaches 104 microns.

Computational algorithm for simulating drug combination schedules*:* Our computational algorithm can be summarized as follows: (i) design an in silico tissue sample for the simulations; (ii) compute forces resulting in the desired fluid and drug supply from the blood capillaries; (iii) compute the interstitial fluid velocity field driven by these forces; (iv) compute compound kinetics (diffusivity,

advection, decay, conversion to an active form). (v) compute cellular uptake of oxygen, pyruvate, and active TH-302 drug, and modify individual cell metabolism depending on levels of accumulated biochemical compounds, for instance: (v-a) if the intracellular level of pyruvate reaches a prescribed threshold, modify model parameters for this host cell to increase its oxygen consumption, (v-b) if the intracellular level of active TH-302 reaches the prescribed threshold, the host cell will be considered dead and will stop consuming the oxygen, pyruvate and drugs. Repeat iteratively these steps to advance the penetration of chemical compounds through the tumor tissue and the tumor tissue response. This model provides a tool to test and analyze various dosing schedules (applied dosage, frequency of treatment, time of treatment supply) in order to find strategies for optimal tumor responses. Thus we will determine dose and timing combinations and ranges that lead to outcomes that can be explored experimentally.

Supplemental References

1. Gillies RJ, Didier N, Denton M. Determination of cell number in monolayer cultures. Anal Biochem. 1986;159:109-13.

2. Wojtkowiak JW, Rothberg JM, Kumar V, Schramm KJ, Haller E, Proemsey JB, et al. Chronic autophagy is a cellular adaptation to tumor acidic pH microenvironments. Cancer Res. 2012;72:3938-47.

3. Rejniak KA, Estrella V, Chen T, Cohen AS, Lloyd MC, Morse DL. The role of tumor tissue architecture in treatment penetration and efficacy: an integrative study. Front Oncol. 2013;3:111.

4. Cortez R. The method of regularized Stokeslets. Siam Journal on Scientific Computing. 2001;23:1204-.

5. Chary SR, Jain RK. Direct measurement of interstitial convection and diffusion of albumin in normal and neoplastic tissues by fluorescence photobleaching, Proc. Nati. Acad. Sci. USA, 1989, 86:5385-5389

6. Chresand TJ, Dale BE, Hanson SL, Gillies RJ. A stirred bath technique for diffusivity measurements in cell matrices. Biotechnology and bioengineering, 1988; 32:1029-1036

7. Jung D, Lin L, Jioa H, Cai X, Duan J-X, Matteucci M, “Pharmacokinetics of TH302: a hypoxially activated prodrug of bromo-isophosphoramide mustard in mice, rats, dogs and monkeys”, Cancer Chemother Pharmacol, 2012 69:643-654

8. Duan JX, Jiao H, Kaizerman J, Stanton T, Evans JW, Lan L, Lorente G, Banica M, Jung D, Wang J, Ma H, Li X, Yang Z, Hoffman RM, Ammons WS, Hart CP, Matteucci M. Potent and highly selective hypoxiaactivated achiral phosphoramidate mustard as anticancer drugs, J. Med. Chem. 2008, 51:2412-2420
